# Atomic Force Microscopy Based Tip-Enhanced Raman Spectroscopy in Biology

**DOI:** 10.3390/ijms19041193

**Published:** 2018-04-13

**Authors:** Lizhen Gao, Huiling Zhao, Tianfeng Li, Peipei Huo, Dong Chen, Bo Liu

**Affiliations:** 1 Institute of Photo-biophysics, School of Physics and Electronics, Henan University, Kaifeng 475004, China; gaolizhen@henu.edu.cn (L.G.); zhl@vip.henu.edu.cn (H.Z.); litianfeng@henu.edu.cn (T.L.); dongchen@henu.edu.cn (D.C.); 2 Laboratory of Functional Molecules and Materials, School of Physics and Optoelectronic Engineering, Shandong University of Technology, Xincun West Road 266, Zibo 255000, China; peipeihuo@stud.edu.cn

**Keywords:** atomic force microscopy based TERS (AFM-TERS), optical diffraction limitation, proteins, nucleic acids, pathogens

## Abstract

Most biological phenomena occur at the nanometer scale, which is not accessible by the conventional optical techniques because of the optical diffraction limitation. Tip-enhanced Raman spectroscopy (TERS), one of the burgeoning probing techniques, not only can provide the topography characterization with high resolution, but also can deliver the chemical or molecular information of a sample beyond the optical diffraction limitation. Therefore, it has been widely used in various structural analyses pertaining to materials science, tissue engineering, biological processes and so on. Based on the different feedback mechanisms, TERS can be classified into three types: atomic force microscopy based TERS system (AFM-TERS), scanning tunneling microscopy based TERS system (STM-TERS) and shear force microscopy based TERS system (SFM-TERS). Among them, AFM-TERS is the most widely adopted feedback system by live biosamples because it can work in liquid and this allows the investigation of biological molecules under native conditions. In this review, we mainly focus on the applications of AFM-TERS in three biological systems: nucleic acids, proteins and pathogens. From the TERS characterization to the data analysis, this review demonstrates that AFM-TERS has great potential applications to visually characterizing the biomolecular structure and crucially detecting more nano-chemical information of biological systems.

## 1. Introduction

Probing the component distribution and the corresponding location mapping of biological macromolecules or biopolymers is one of the fascinating research fields because it is crucial to inquire and understand the novel properties of vital materials in the living body, even in the remediation of tissues and the therapy of many diseases [1,2,3,4]. For example, Nucleic acids, a category of biological macromolecular compounds, can be synthesized through polymerizing nucleotides with different stoichiometric coefficients and different arrangements. They play important roles in storing and transmitting genetic information during numerous biological processes including replication and synthesis of proteins, heredity and variation of life phenomena, and so on. Therefore, detecting nucleotide sequences and mapping their corresponding locations are of great importance in life science and biomedical technology. Nowadays, there are many methods including the Maxam-Gilbert sequencing and Chain-termination techniques which have been developed to determine DNA sequences and investigate the structure and function of nucleic acids [5,6,7]. Especially, the configuration, rotation period, major groove and minor groove of DNA molecule were firstly probed at the atomic level by scanning tunneling microscopy (STM) in 1989. It provided a visual and reliable demonstration of the double-helix of DNA molecules [8]. However, detecting the molecular characterization and its correlative structural information is still a challenge in the last decades. 

Except for DNA and RNA, proteins are another kind of biological macromolecules which are consisted of one or more long chains of amino acid residues. Each kind of protein molecule has a unique three-dimensional structure and carries out diversified biological functions in the living body. According to the difference of amino acid sequences, proteins can also be divided into several categories such as Antibody, Enzyme, Messenger, Transport and Storage etc. [9]. Undoubtedly, discovering simultaneously the tertiary structure and corresponding amino acid sequence of a protein can provide important clues to how a protein performs its function [10,11]. Even more, these related investigations will not only contribute to the understanding of vital biological processes, but also be beneficial to the design of new ligands applied to the development of new drug molecules. However, many traditional analysis techniques (scanning electron microscopy, scanning probe microscopy, X-ray diffraction, nuclear magnetic resonance, etc.) usually can only provide the structural or chemical information [12,13,14,15]. Therefore, a tool which can combine topography with chemical information will accelerate the research of proteins. 

Tip enhanced Raman spectroscopy (TERS), a newly-developed technique combining scanning probe microscopy (SPM) with the Raman spectroscopy together, can provide chemical analysis and structural topography of a sample with a high spatial resolution [16,17]. In TERS experiment, the Raman signal of molecules over the sample surface is enhanced due to the coefficient of the surface plasmonic resonance and the lighting-rod effect. When a probe scans over the sample surface at a certain speed, the topography at a resolution better than 20 nm and the label-free Raman mapping can be obtained simultaneously. Besides, atomic force microscopy based TERS (AFM-TERS) inherit two advantages from AFM. Firstly, AFM can be operated in liquid conditions, which makes it possible to study biological samples in native environments [18,19,20]. Secondly, AFM may be used in two different operating modes, namely the contact mode and the tapping mode. When the fragile samples, such as RNAs [21] and lipid bilayers [22], are investigated, the tapping mode can be chosen to avoid the damage to the sample since the force between the tip and the sample is smaller in this mode. Due to these reasons, AFM-TERS is gradually employed to investigate masses of biological samples, such as nucleic acids [23], proteins [24,25], pathogens [26], lipids and cell membranes [27]. 

In this review, the working principle of the TERS setup will be presented and discussed. Then the applications of AFM-TERS in nucleic acids, proteins and pathogens will be presented. All of these results indicate that the simultaneous acquisition of the structural and chemical information at the same scanning point of a sample through AFM-TERS technique can not only help to understand the relationship between the structure, composition and function of a biomolecule, but also visually clarify the variation mechanism of lives in different physiological or pathological conditions, thus giving an important guidance to future pharmaceutical sciences.

## 2. Tip Enhanced Raman Spectroscopy (TERS) Experiment

TERS technique is a combination technique composed of SPM, an illumination source and Raman spectroscopy. In TERS, the feedback system of SPM makes sure that the metal or metallized tip remains very close to a sample. When an incident laser with a proper wavelength is focused onto the tip apex, a strong localized electromagnetic field can be obtained between SPM tip and the sample surface. Then the optical properties with a resolution surpassing the diffraction limit and the topographic characteristics of the sample are detected simultaneously. The basic structure and working principle of this technique will be described below.

### 2.1. Optical Geometries

**Figure 1:**
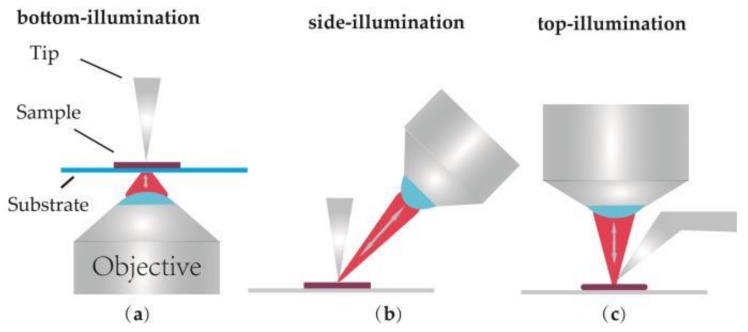
Illustration of commonly used illumination and detection geometries for Tip-enhanced Raman spectroscopy (TERS). (**a**) Bottom-illumination, (**b**) Side-illumination, (**c**) Top-illumination.

According to the lighting direction, the optical geometries of TERS equipment can be divided into three types: bottom illumination, top illumination and side illumination, as shown in Figure 1. Figure 1a sketches the bottom illumination which is composed of an inverted objective and a scanning system. This type is also called transmission mode. In this mode, the end of the TERS probe can be illuminated and enhanced efficiently by an inverted oil immersion objective (N.A. = 1.4) [28,29,30]. Such a TERS system was employed by Hayazawa et al. In the related experiments, an AFM-TERS system with a silver-coated tip and an inverted oil immersion objective was used to collect TERS information of the samples [31,32,33]. It is obvious that an opaque sample is unsuitable since the laser beam cannot pass through the sample and illuminate the end of the tip. Therefore, this kind of TERS system was used during the early development stages of TERS. 

In order to avoid this drawback, side-illumination (Figure 1b) and top-illumination (Figure 1c) were gradually developed [34,35,36,37,38]. In these two kinds of TERS setups, objectives located above the sample and long working distance objectives (N.A. = 0.28–0.7) are required to focus laser onto the sample surface. These two modes are simply named reflection mode, corresponding to the previous transmission mode. In side-illumination, signal loss is always induced by the shadow of the tip since there is an angle between the objective and the vertical tip. Laser with higher power is required to compensate the lower collection efficiency. Top-illumination can offer a no-loss signal, but a top visual cantilever (shown in Figure 1c) should be used in order to avoid the shadowing effect of the cantilever. Presently, the flexible characterization of these two TERS modes makes them widely applied in the study of CNTs [31,39,40], graphenes [41,42], biological macromolecules [38,43,44] and other opaque samples [45,46]. Finally, the advantage and disadvantage of the different optical geometries are summarized and listed in Table 1.

### 2.2. Feedback Systems

**Figure 2:**
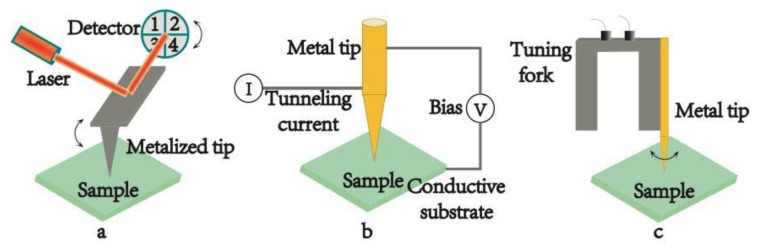
Schematic diagrams of the different scanning probe microscopy (SPM) feedback systems used in TERS. (**a**) atomic force microscopy (AFM) feedback system, (**b**) scanning tunneling microscopy (STM) feedback system, (**c**) shear force microscopy (SFM) feedback system.

According to the feedback mechanisms which keep the tip close to the sample surface, TERS systems are generally divided into three types: AFM-TERS, STM-TERS and shear force microscopy based TERS (SFM-TERS). They are schemed in Figure 2a–c, respectively.

#### 2.2.1. Atomic Force Microscopy Based Tip Enhanced Raman Spectrum (AFM-TERS)

In AFM, the interaction between the tip and the sample is transduced into the motion of a small spring-like cantilever because the tip is fixed to the end of cantilever. A quadruple photo-detector records the motion via a semiconductor laser, which is reflected onto the detector from the back of an AFM cantilever, as shown in Figure 2a. The tips used in AFM-TERS are usually the commercial silicon or silicon nitride tips covered with metal layers. The range of the plasmon resonance can be controlled through changing the different coating metal layer from gold (Au) or silver (Ag) to aluminum (Al). Au possesses strong enhancement in the red spectral region. Ag shows stronger signal in the blue-green spectral region and in the UV and deep UV spectral regions; Al shows promising enhancement, which makes it possible to study the electronic resonance of different biological samples by TERS [47]. Furthermore, the thickness of the metal coating layer can also be controlled by experimental parameters, so one can seek for the optimal combination condition of Raman enhancement and image resolution by tuning the size of the tip [48]. In addition, the metallized tip can offer a strong hot spot by artificially adding some special nanostructures on the probe apex. In this case the metal substrate is not required, so the sample can be placed on a glass substrate and the tip apex can be illuminated in the bottom-illumination mode. The major advantage of this geometry is that the short-working-distance high-NA objective lens of AFM-TERS make the apex of the metal tip illuminated effectively [31,32,33]. Except for the superiorities of the probe, AFM-TERS can be operated in liquid conditions [18,19,20]. It is of great significance to the development of life science, for it allows one to study biological samples in native conditions. More and more results proved that AFM-TERS could keep biological samples alive and continue their physiological activities during the measurements in liquid environment, particularly in water. Given the versatile characterization, many home-made and commercial TERS systems have adopted the AFM-based TERS setup [49]. This prompts AFM-TERS system to become a powerful tool for characterizing various materials from single molecules [50,51], biological materials to low dimensional nanomaterials [39,42]. 

#### 2.2.2. Scanning Tunneling Microscopy Based Tip Enhanced Raman Spectrum (STM-TERS)

The surface height and density of states of the sample in STM are mapped by the tunneling current, which is controlled by a voltage bias between probe and substrate (see Figure 2b). In the system, the tips are made of solid metal, usually gold. In order to generate a tunneling current, conductive or semi-conductive substrates must be used and the distance between the tip and the sample surface should be less than 1 nm [45]. All these factors are beneficial to the enhancement of the optical signal. STM-TERS has a higher spatial resolution which is related to the STM technique. It can operate under high-vacuum and low-temperature measurements [52], which is in favor of better signal stability of the sample. In addition, STM-TERS can also be manipulated in liquid. A few studies have been carried out by electrochemical STM-TERS (EC-TERS) [53,54,55,56], where the STM probe was immersed in some suitable solvent for the electrochemical measurement. In EC-TERS, a suitable liquid is primarily required to maintain the tunneling current, and meanwhile a proper coating on the tip should be satisfied to ensure that the Faradaic current doesn’t interfere with the tunneling current. However, in most cases the solvents used are not suitable for keeping the physiological activities of biological samples. Therefore, no TERS image of biological samples in STM-based liquid TERS has been reported so far. Besides, the opaque thick metallic substrates make it impossible to use the transmission mode in STM-TERS.

#### 2.2.3. Shear Force Microscopy Based Tip Enhanced Raman Spectrum (SFM-TERS)

SFM-TERS is the third kind of TERS technique in which a metal tip is mounted onto the prong of a quartz tuning fork (see Figure 2c). All the properties of SFM-TERS system are similar to those of AFM-TERS system, except that SFM system is controlled by a shear force during the scanning process. Both the bottom-illumination mode and the side-illumination mode can be used in SFM-TERS. Although SFM-TERS was developed relatively late and it was only employed by a few groups, remarkable research results have been shown by the technique [57,58,59].

Hereto, the characteristics of the three different feedback systems are shown in Table 2.

### 2.3. Tip Preparation

The preparation of the TERS probes is a primary and vital step during the TERS experiments since it influences directly on the spatial resolution, reproducibility, and enhancement of the chemical signal originating from the sample surface. As discussed above, there are different kinds of tips corresponding to the different feedback systems used in TERS technique. The metal tips used in STM-TERS and SFM-TERS are usually prepared by the electrochemical etching methods [60,61]. As for the probes of AFM-TERS, they can be obtained by covering the commercial probes of silicon or silicon nitride with silver or gold layers [62,63,64,65]. Silver is known as an ideal material to generate high local surface plasmons covering the entire visible light region [66,67]. However, a probe coated with silver layer is difficult to keep activity in a long time since silver can be oxidized easily in atmosphere [68]. The probe coated with gold layer is chemically inert to oxidation, but it can generate high signal enhancement only in the red region. In the following section, we will focus on the varieties of methods to prepare the tips used in AFM-TERS measurements. 

#### 2.3.1. Chemical Vapor Deposition

Chemical vapor deposition (CVD) is a traditional method to produce metallized TERS probe. By this method, many hemispherical metal islands are deposited on the surface of a probe [31,39,49]. The islands on the apex of the probe will offer an enhanced electromagnetic field during the TERS measurement. However, the application of the TERS tip is restricted by its low yield and low reproducibility caused by the random nucleation and the growth process of metal nanoparticles around the tip-apex [69,70].

#### 2.3.2. Mirror Reaction

Mirror reaction is a facile method of depositing smoothly packed particles on silica or other substrates like silicon nitride. In the mirror reaction, silver spheres with controllable size can be tightly arranged on the silicon or silicon nitride probe just by changing the concentration of the silver nitrate solution [71,72]. Therefore, the metallized probe can be easily prepared by the mirror reaction. However, some contaminations might be produced in this chemical reaction and they can degrade the enhancement efficiency of the tip.

In addition, there are other methods including ion beam lithography [73,74], vapor-liquid-solid [75,76], tip on tip [77], template-stripping [78] and so on. However, all of the methods reviewed here bear their own challenges and have some room for improvement. 

In contrast to the metal tips used in STM-TERS or SFM-TERS, AFM probes coated with metal layers suffer from low resolution and limited active time because the metal cover may increase the apex size of the probe and wear off during the measurements. Metal nanowire attached to a tip is another alternative in AFM-TERS [79]. It has been demonstrated that such massive TERS active metal probes can improve spatial resolution, give a higher light coupling efficiency and prolong the propagation length of the surface plasmons.

### 2.4. Gap Mode

**Figure 3:**
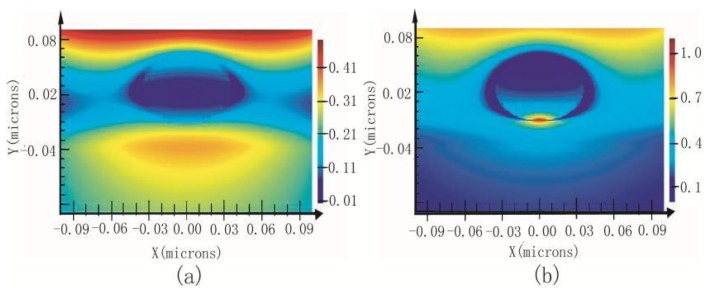
Electromagnetic field between 70 nm gold nanoparticles and different substrates; (**a**) silicon, (**b**) Au.

If a metallized probe is brought very close to a substrate with gold layer, there will arise a stronger enhancement signal owning to the sandwich-type assay called “gap mode”. The signal strength is mainly attributed to the particle dimension, the gap dimension and the polarization of the irradiating field [80]. A few experiments were carried out to investigate C_60_ molecules in different diluted solutions at the nanomolar scale. The results indicated that the gap mode spectrum had higher signal intensity than SERS spectrum [81]. This conclusion is the same with the calculation results of Finite-Difference Time-Domain (FDTD), which is a numerical analysis technique used for modeling computational electrodynamics. Since it is a time-domain method, FDTD solution can cover a wide frequency range with a single simulation run, and treat nonlinear material properties in a natural way. Figure 3 shows the FDTD results of the electromagnetic field between 70 nm gold nanoparticles and different substrates. Distance between the particle and corresponding substrate is adjusted to 1 nm. The substrate in Figure 3a is silicon and that in Figure 3b is gold. The enhanced area in Figure 3a is about 70 nm (similar to the diameter of the particle) and the enhanced area in Figure 3b is about 10 nm with a stronger electromagnetic field (typical gap mode). This proves that gap mode can make signal intensities and resolution improved at the same time for it can confine stronger light within a region that is much smaller than the diameter of the tip.

## 3. Applications of AFM-TERS in Biology

How to demonstrate the relationship between the structure, function and chemical information of biological molecules in natural environments has always been one of the big challenges in life science. AFM-TERS can provide both the structure and chemical information of a sample at the nanoscale simultaneously in various experimental conditions, which causes AFM-TERS to be widely used in biological samples such as nucleic acids, proteins, pathogens, lipids and cell membranes, etc.

### 3.1. Nucleic Acids

TERS technique has been widely applied to characterize nucleic acid bases due to its characteristic of small sample amount, high sensitivity and direct-sequencing [19,82,83,84]. Five nucleic acid bases including adenine (A), cytosine(C), thymine (T), guanine (G), and uracil (U) were involved in such analysis. The earlier TERS investigations were focused on the nucleic acid nanocrystals [85,86,87,88]. For example, Regina Treffer et al. demonstrated the reproducibility of AFM-TERS by investigating the single-stranded adenine and uracil homopolymers on the different kinds of substrates. Later, the same equipment was used to study single-stranded calf thymus DNA with arbitrary sequence [89]. The result proved that it was possible to distinguish different nucleobases clearly. The combination of sensitivity and reproducibility shows that TERS can meet the crucial demands for a sequencing procedure. Subsequently, a series of successful TERS investigations of single-stranded RNA, single-stranded DNA and double-stranded DNA have come forth. 

**Figure 4:**
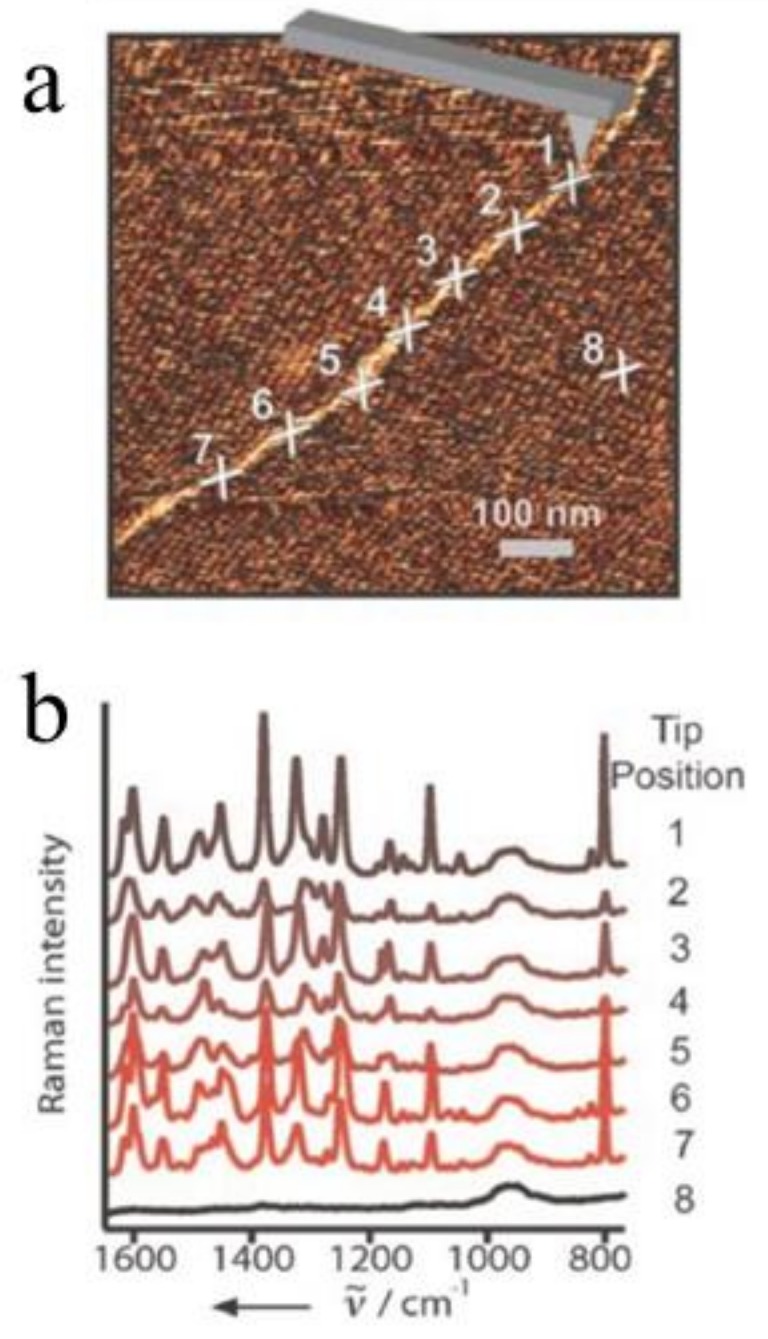
TERS experiment along an RNA strand. (**a**) Topography image (same as in Figure 2a) showing seven adjacent spots corresponding to the positions of the TERS experiments and one additional spot for the reference measurement (position 8); (**b**) The Ramans pectra of positions in (**a**). Reprinted with permission from ref. [44]. Copyright 2008, Wiley-VCH Verlag GmbH & Co. KGaA, Weinheim, Germany.

E. Bailo and V. Deckert presented a TERS experiment for sequencing a single-stranded RNA of cytosine(poly(C)) in nanomolecular quantities [44]. With an Ag-covered AFM tip, the topography of a single-strand RNA was imaged (Figure 4a) and then eight Raman spectra (including seven from different positions along the RNA chain and one from the reference) were measured by TERS (Figure 4b). According to the article, the signal-to-noise ratio (SNR) of the TERS spectrum was obtained by dividing the intensity of the most intense Raman band by two-times the standard deviation of the noise level measured in a signal-free section of the spectrum. The SNR here was about 200. Every TERS spectrum exhibited 30–60 bases beneath the tip (about 20 nm) and each base contributed a SNR about 3–7 to the spectrum. This indicated that the visualized image with high contrast for the direct-sequencing of nucleic acid became feasible. 

Najjar et al. presented another TERS experiment to investigate combed double-stranded DNA bundles [90]. With a tip covered by a Ag/Au bilayer, all of the DNA nucleobases and those backbones containing phosphodiester and sugar groups could be identified by the Raman signal of TERS. This indicated that DNA hybridization could be detected via the TERS technique. 

Lipiec et al. characterized the molecular structure of double strand breaks (DSBs) by the TERS technique [23]. The controlled and irradiated samples were firstly imaged by the AFM technique, which showed that the plasmids went into a relaxed state or a linear shape instead of their supercoiled quaternary structure following ultraviolet C (UVC) exposure. Then, potential cleavage sites were found according to the TERS spectra. Comparing these images, it could be concluded that the DSBs mainly resulted from the 3′- and 5′-bonds of deoxyribose upon UVC-exposure and the 3′-bond was the most likely type of DSBs since it accounted for more than half of the breaks detected.

The examples above show that AFM-TERS has succeeded in distinguishing the respective nucleobases, detecting DNA hybridization and characterizing the molecular structure of DSBs. AFM-TERS permits chemical identification at a scale of 10 nm (which is smaller than the size of the tip apex radius). It means there are 30–60 bases responsible for the Raman signal in every spectrum. The resolution is not enough to unambiguously distinguish a single base. It’s well known that the resolution of AFM increases with the decrease in the size of the scanning tip apex. However, for AFM-TERS, decreasing the size of apex means a thinner metal layer on AFM tip. As a result, the tip might lose its TERS activity. In order to increase the resolution of AFM-TERS, metal nanowire attached to a tip is another alternative in AFM-TERS. However, the related work has not been published yet. Recently, Rui Zhang et al. reported the outstanding capability of STM-TERS to distinguish individual DNA bases (adenine and thymine) with a super-high spatial resolution of ~0.9 nm [57]. This work opened a new way to achieve single-molecule DNA sequencing on surfaces by TERS technique. It should be noted that the experiments were carried out under the condition of low temperature and high vacuum. Obviously STM-TERS has a higher resolution than AFM-TERS not only because of the massive metal probes, but because of the extreme conditions. However, it is impossible for the biological samples to keep alive under such extreme conditions.

### 3.2. Proteins

Proteins are the executors of physiological functions and the direct embodiments of life phenomena. The study of the protein structure and function will visually clarify the variation mechanisms of lives in different physiological or pathological conditions. 

The heme protein cytochrome c (Cc), consisting of heme, covalently bound to the protein through thioether links and a Fe-amino coordination, is one of the proteins which was investigated by TERS in the early stage. In this specific experiment, Raman properties of Cc were detected by AFM-TERS and SERS at the same time [91]. The result showed that with an Ag coated AFM-tip, TERS spectra could present the Raman signal of both the heme and amino acids Cc. However, the information of amino acids could not be observed in SERS. This proved that with superior spatial resolution and stronger enhancements, TERS could give more structural information about large biomolecules compared with SERS. 

Additionally, a series of contrast experiments about bovine serum albumin (BSA), phenylalanine and tyrosine were carried out. In the experiments, Raman signals originating from conventional Raman, SERS and TERS at the same excitation wavelength were compared [92]. It was found that the relative spectral intensities of the Raman peaks were usually different, but the positions of them were unchanged in all these three methods. These research results demonstrated that TERS characterization could not only yield spectroscopic fingerprints similar to conventional Raman and SERS, but provide a higher spatial resolution at nanometer scale. The information of TERS would provide more useful guidelines for understanding the structures and functions of biological molecules.

**Figure 5:**
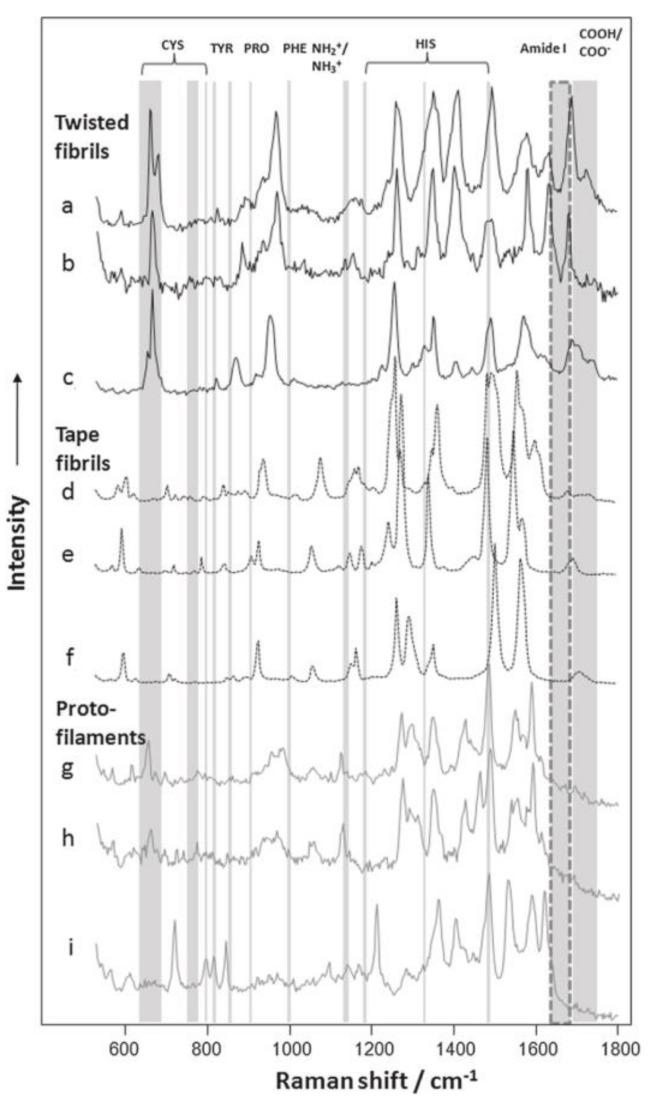
Selected TERS spectra acquired from the surfaces of insulin fibril polymorphs and their protofilaments. TERS spectra collected from the surfaces of twisted are shown in a–c and d–f are the Raman signals of flat fibrils, g–i are the TERS spectra collected from the surfaces of their protofilaments. Specific amino acid vibrational modes are marked with gray lines. Amide I bands are marked with dashed lines, showing the borders between protein secondary structures. NH2/NH3 (1144 cm^−1^) and COOH/COO—(1687–1700 cm^−1^) vibrational modes are also indicated. Reprinted with permission from ref. [25]. Copyright 2014, the Biophysical Society.

With these incomparable advantages, more and more complex protein structures were analyzed by TERS technique. For example, amyloid fibrils, caused by protein aggregation inside the human body, have been acknowledged to be responsible for many neurodegenerative diseases, such as Alzheimer’s disease, Huntington’s disease and prion diseases. In the early years, it could not be fully understood what caused the amyloid-like protein aggregations. Recent researches on topography and chemical characterization detected by TERS offered much useful information [93,94,95,96]. One of the typical TERS experiments related with amyloid fibers is about insulin protofilaments, fibril polymorphs and their topographies. In the experiment, the surfaces of two insulin fibril polymorphs with flat and twisted morphologies were investigated. TERS spectra showed that (see Figure 5) [25] both twisted fibrils and flat fibrils had different types of amino acid residues on their surfaces. Cys was potentially involved in the form of the twisted fibrils, but it was most likely nothing to do with the propagation of the flat fibrils. These results demonstrated that there were different formation mechanisms for twisted fibrils and flat fibrils.

These above results present the plausible use of TERS technique to characterize surface amino acid components and protein secondary structures, which are helpful to understand the mechanisms of fibril growth and propagation. Therefore, TERS characterization can be widely used to define the keys to preventing or slowing down the progression of various neurodegenerative diseases.

### 3.3. Pathogens

Successfully detecting the structure, dynamics and function of pathogens is crucial for public health since these works can provide some guidelines on the prevention, diagnosis and therapy of the diseases. 

The first kind of pathogens detected by AFM-TERS was Gram-positive bacteria Staphylococcus epidermis [43]. With the AFM-TERS technique, the topography of a single epidermis cell and AFM-TERS spectra detected at the different positions of the bacterial surface were investigated. The results indicated that most of Raman bands were originated from peptides and polysaccharides, which are well known as the bacterial surface components [97]. This demonstrated that TERS can be used to investigate the more complex biological systems such as bacteria.

Another previous AFM-TERS research of pathogens was tobacco mosaic virus (TMV) which appeared as a rod-like capsid made by one structural protein and one single RNA molecule. In this work, the rod-like structure of a single TMV particle was characterized and four TERS spectra at different positions were obtained through approaching the TERS tip onto the sample surface [98]. The results indicated that there were some slight differences in the four Raman bands corresponding to the coat protein and RNA of the single TMV. These differences were attributed to the different orientations of the molecules, and might also be caused by moving the TERS probe near to the surface of the sample from one point to another.

**Figure 6:**
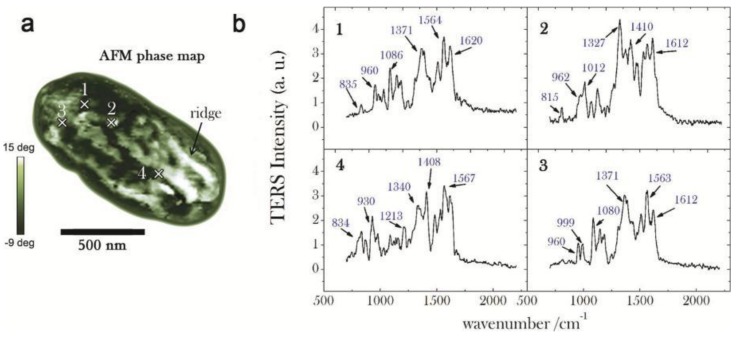
(**a**) AFM phase map of a whole spore acquired in tapping mode (scale bar = 500 nm, step-size = 20 nm): bright (dark) zones correspond to a positive (negative) phase-lag, namely to regions of higher (lower) stiffness; (**b**) Near-field (normalized) TERS spectra acquired in four selected positions over the spore coat, corresponding to the cross-marks in (**a**): in order to emphasize the relevant Raman features, background signal is identified by a fourth-order polynomial fitting routine and consequently subtracted; reprinted with permission from reference [99]; Copyright 2014 American Chemical Society.

In recent years, Bacillus subtilis spores were also investigated by combining TERS with principal components analysis (PCA) [99]. In this work, the TERS mapping of the spore was analyzed, which provided some important information about the component distribution of the spore ridges (see Figure 6). These results showed the first and crucial step toward the correlative surface-chemistry characterization of spores via nanoscale spectroscopy. The authors further demonstrated that TERS mapping can be connected to AFM-phase images by the combination of PCA and TERS, which could allow one to visually discriminate the relevant chemical distribution of complex biological systems.

With the characteristics of high sensitivity and superior spatial resolution, the application of AFM-TERS in biology has been increasing rapidly. Table 3 exhibits the structures and detection range of the three biomolecules characterized by AFM-TERS techniques. These results demonstrate that the TERS technique moves further into the direct detection of biomolecules at the nanolevel. However, it is also obvious that the more complex biological molecular structure, the less information can be detected in TERS test. Undoubtedly, this indicates that there is much room for improvement in applying AFM-TERS in biology. 

## 4. Conclusions

One of the ultimate targets in the study of life science is to seek for the relationship between the structure, function and dynamics of biomolecules in the natural environment. This needs setups which can provide the structural information and corresponding chemical information of samples with a higher resolution. In this view, we have presented AFM-TERS as a technique, which can provide topography and corresponding optical information at a nanoscale resolution at the same time. The outstanding performance makes it an important approach for the study of biochemical species such as nucleic acids, proteins, bacteria, viruses and so on. With a metallized probe, a Raman image with a spatial resolution in the 10 nm range can be obtained by AFM-TERS, it opens a new way in DNA direct-sequencing. Beyond that, it can be used to provide the structural images and label-free chemical information of proteins and pathogens. Those relevant works proved that AFM-TERS could be used to investigate the more complex biological systems and their results could offer beneficial guidelines for biomedical research. For example, AFM-TERS with a sub-10 nm resolution can detect the diseases related to the mutations of DNA [23,44,90]. AFM-TERS can also be used to characterize amyloid fibrils, which can be helpful to seek for the reasons of various neurodegenerative diseases [25]. Furthermore, biomarkers are biochemical indexes which can be used to measure and evaluate the specific features in biological processes, pathogenic processes, and management processes [100,101,102]. Detecting diagnostic markers can help to the identification, diagnosis, remedy and prevention of diseases because it can mark the changes in the structures and functions of organs, tissues, and cells. The markers include various molecules from inorganic salts to biomolecules (such as DNA, RNA, amino acid, protein etc.), which can be characterized by AFM-TERS. AFM-TERS may become a new analysis method for biomarkers in the near future. All above examples prove that AFM-TERS has the potential to pre-diagnose some clinical diseases and shed light on the pathogenesis of diseases at the molecular level. 

In recent years, significant progress has been made in the study of biological materials by AFM-TERS. However, further optimization of the TERS technique is necessary in order to make it suitable for a wide range. For example, studying biomolecules in liquid is crucial since their physiological activities can be kept and detected only in native environments. However, AFM-TERS in liquid just emerges and is in the urgent need of being improved. On the other hand, AFM-TERS tips with a more pronounced performance enhancement should be made in order to avoid biological samples damaged by the high powerful laser. In addition, this system is expected to be developed in the characterization of more soft biological samples.

## Figures and Tables

**Table 1:** The advantage and disadvantage of the different optical geometries.

Optical Geometries	Bottom-Illumination	Side-Illumination	Top-Illumination
Advantage	Efficient enhancement	Wide application	Wide application
Disadvantage	Only for transparent simple	Signal loss	top visual cantilevers should be used

**Table 2:** Characteristics of the different feedback system.

Feedback System	AFM-TERS	STM-TERS	SFM-TERS
controller	Atomic force	tunneling current	Shear force
Tip material	silicon or silicon nitride tip covered with metal	metal	metal
optical geometries	reflection mode and transmission mode	reflection mode	reflection mode and transmission mode
substrate	all kinds of substrates [39,40,41,42,43,44,45,46]	conductive or semi-conductive substrates	all kinds of substrates
environment	Gas [39,40,41,42,43,44,45,46], liquid [49,50,51]	gas, liquid(special) [53,54,55,56], high-vacuum, low-temperature [52]	gas

**Table 3:** The structure of the nucleic acid, protein and pathogen and their border of AFM-TERS had been detected by TERS techniques.

Biomolecule	Nucleic Acid	Protein	Pathogen
Structure	A single- or double-stranded structure consisted of five nucleotides	Three-dimensional structure consisted of one or more long chains of amino acid residues	RNAs and Proteins
AFM-TERS test	direct-sequencing; DNA hybridization; DNA double strand breaks [19,44,82,83,84,85,86,87,88,89]	Conformal changes and amino acid distributions [25,90,91,92,93,94,95,96]	Peptides and polysaccharides [43,98,99]

## References

[1] Dieluweit S., Csiszr A., Rubner W., Fleischhauer J., Houben S., Merkel R. (2010). Mechanical properties of bare and protein-coated giant unilamellar phospholipid vesicles. A comparative study of micropipet aspiration and atomic force microscopy. Langmuir.

[2] Santos E.B., Morris J.K., Glynos E., Sboros V., Koutsos V. (2012). Nanomechanical properties of phospholipid microbubbles. Langmuir.

[3] Sirghi L. (2010). Atomic Force Microscopy Indentation of Living Cells.

[4] Franz V., Loi S., Mller H., Bamberg E., Butt H.J. (2002). Tip penetration through lipid bilayers in atomic force microscopy. Colloids Surf. B Biointerfaces.

[5] Gilbert W. (1981). DNA sequencing and gene structure. Biosci. Rep..

[6] Sanger F., Nicklen S., Coulson A.R. (1992). DNA sequencing with chain-terminating inhibitors 1977. Biotechnology.

[7] Gilbert W. (1977). A new method for sequencing DNA. Proc. Natl. Acad. Sci. USA.

[8] Lindsay S.M., Nagahara L.A., Thundat T., Oden P. (1989). Sequence, packing and nanometer scale structure in STM images of nucleic acids under water. J. Biomol. Struct. Dyn..

[9] Gutteridge A., Thornton J.M. (2005). Understanding nature’s catalytic toolkit. Trends Biochem. Sci..

[10] Copland J.A., Sheffield-Moore M., Kodzic-Zivanovic N., Gentry S., Lamprou G., Tzortzatou-Stathopoulou F., Zoumpourlis V., Urban R.J., Vlahopoulos S.A. (2009). Sex steroid receptors in skeletal differentiation and epithelial neoplasia: Is tissue-specific intervention possible. Bioessays.

[11] Samarin S., Nusrat A. (2009). Regulation of epithelial apical junctional complex by Rho family GTPases. Front. Biosci..

[12] Gonen T., Cheng Y.F., Sliz P., Hiroaki Y., Fujiyoshi Y., Harrison S.C., Walz T. (2005). Lipid-protein interactions in double-layered two-dimensional AQP0 crystals. Nature.

[13] Standley D.M., Kinjo A.R., Kinoshita K., Nakamura H. (2008). Protein structure databases with new web services for structural biology and biomedical research. Brief. Bioinform..

[14] Walian P., Cross T.A., Jap B.K. (2004). Structural genomics of membrane proteins. Genome Biol..

[15] Sleator R.D., Walsh P. (2010). An overview of in silico protein function prediction. Arch. Microbiol..

[16] Garcia-Parajo M.F. (2008). Optical antennas focus in on biology. Nat. Photonics.

[17] Huang B., Bates M., Zhuang X.W. (2009). Super-Resolution Fluorescence Microscopy. Annu. Rev. Biochem..

[18] Schmid T., Yeo B.-S., Leong G., Stadler J., Zenobi R. (2009). Performing Tip-Enhanced Raman Spectroscopy in Liquids. J. Raman Spectrosc..

[19] Yeo B.S., Stadler J., Schmid T., Zenobi R., Zhang W.H. (2009). Tip Enhanced Raman Spectroscopy—Its Status, Challenges and Future Directions. Chem. Phys. Lett..

[20] Nakata A., Nomoto T., Toyota T., Fujinami M. (2013). Tip-enhanced Raman Spectroscopy of Lipid Bilayers in Water with an Alumina- and Silver-coated Tungsten Tip. Anal. Sci..

[21] Kasas S., Thomson N.H., Smith B.L., Hansma H.G., Zhu X., Guthold M., Bustamante C., Kool E.T., Kashlev M., Hansma P.K. (1997). *Escherichia coli* RNA polymerase activity observed using atomic force microscopy. Biochemistry.

[22] Muhammad S.K., Noura S.D., Ghulam M., Darayas P., Bakhrom B., John D.W. (2017). Electrophysiology of Epithelial Sodium Channel (ENaC) Embedded in Supported Lipid Bilayer Using a Single Nanopore Chip. Langmuir.

[23] Lipiec E., Sekine R., Bielecki J., Kwiatek W.M., Wood B.R. (2014). Molecular Characterization of DNA Double Strand Breaks with Tip-Enhanced Raman Scattering. Angew. Chem. Int. Ed..

[24] Deckert-Gaudig T., Bailo E., Deckert V. (2009). Tip-enhanced Raman scattering (TERS) of oxidised glutathione on an ultraflat gold nanoplate. Phys. Chem. Chem. Phys..

[25] Kurouski D., Deckert-Gaudig T., Deckert V., Lednev I.K. (2014). Surface Characterization of Insulin Protofilaments and Fibril Polymorphs Using Tip-Enhanced Raman Spectroscopy (TERS). Biophys. J..

[26] Wood B.R., Bailo E., Khiavi M.A., Tilley L., Deed S., Deckert-Gaudig T., McNaughton D., Deckert V. (2011). Tip-Enhanced Raman Scattering (TERS) from Hemozoin Crystals within a Sectioned Erythrocyte. Nano Lett..

[27] Bohme R., Richter M., Cialla D., Rosch P., Deckert V., Popp J. (2009). Towards a specific characterisation of components on a cell surface—Combined TERS-investigations of lipids and human cells. J. Raman Spectrosc..

[28] Kumar N., Rae A., Roy D. (2014). Accurate measurement of enhancement factor in tip-enhanced Raman spectroscopy through elimination of far-field artefacts. Appl. Phys. Lett..

[29] Lerman G.M., Levy U. (2008). Effect of radial polarization and apodization on spot size under tight focusing conditions. Opt. Express.

[30] Wang J., Wu X., Wang R., Zhang M., Marulanda J.M. (2011). Detection of Carbon Nanotubes Using Tip-Enhanced Raman Spectroscopy. Electronic Properties of Carbon Nanotubes.

[31] Hayazawa N., Inouye Y., Sekkat Z., Kawata S. (2000). Metallized tip amplification of near-field Raman scattering. Opt. Commun..

[32] Hayazawa N. (2001). Near-field Raman scattering enhanced by ametallized tip. Chem. Phys. Lett..

[33] Hayazawa N. (2002). Near-field Raman imaging of organic mole-cules by an apertureless metallic probe scanning optical microscope. J. Chem. Phys..

[34] Mehtani J.D. (2005). Nano-Raman spectroscopy with side-illumination optics. J. Raman Spectrosc..

[35] Saito Y., Motohashi M., Hayazawa N., Iyoki M., Kawata S. (2006). Nanoscale characterization of strained silicon by tip-enhanced Raman spectroscope in reflection mode. Appl. Phys. Lett..

[36] Stadler J., Schmid T., Zenobi R. (2010). Nanoscale chemical imaging using top-illumination tip-enhanced Raman spectroscopy. Nano Lett..

[37] Rodriguez R.D. (2012). Compact metal probes: A solution for atomic force microscopy based tip-enhanced Raman spectroscopy. Rev. Sci. Instrum..

[38] Naumenko D., Snitka V., Serviene E., Bruzaite I., Snopok B. (2013). In vivo characterization of protein uptake by yeast cell envelope: Single cell AFM imaging and μ-tipenhanced Raman scattering study. Analyst.

[39] Yu J., Saito Y., Ichimura T., Kawata S., Verma P. (2013). Far-field free tapping-mode tip-enhanced Raman microscopy. Appl. Phys. Lett..

[40] Anderson M.S. (2000). Locally enhanced Raman spectroscopy with an atomic force microscope. Appl. Phys. Lett..

[41] Rickman R.H., Dunstan P.R. (2014). Enhancement of lattice defect signatures in graphene and ultrathin graphite using tip-enhanced Raman spectroscopy. J. Raman Spectrosc..

[42] Ghislandi M., Hoffmann G.G., Tkalya E., Xue L.J., De With G. (2012). Tip-Enhanced Raman Spectroscopy and Mapping of Graphene Sheets. Appl. Spectrosc. Rev..

[43] Neugebauer U., Rosch P., Schmitt M., Popp J., Julien C., Rasmussen A., Budich C., Deckert V. (2006). On the way to nanometer-sized information of the bacterial surface by tip-enhanced Raman spectroscopy. Chemphyschem.

[44] Bailo E., Deckert V. (2008). Tip-enhanced Raman spectroscopy of single RNA strands: Towards a novel direct-sequencing method. Angew. Chem. Int. Ed..

[45] Michaels A.M., Jiang J., Brus L. (2000). Ag nanocrystal junctions as the site for surface-enhanced Raman scattering of single Rhodamine 6G molecules. J. Phys. Chem. B.

[46] Williams C., Roy D. (2008). Fabrication of gold tips suitable for tip-enhanced Raman spectroscopy. J. Vac. Sci. Technol. B.

[47] Taguchi A., Hayazawa N., Furusawa K., Ishitobi H., Kawata S. (2009). Deep-UV tip-enhanced Raman scattering. J. Raman Spectrosc..

[48] Meng L.Y., Huang T.X., Wang X., Chen S., Yang Z.L., Ren B. (2015). Gold-coated AFM tips for tip-enhanced Raman spectroscopy: Theoretical calculation and experimental demonstration. Opt. Express.

[49] Stockle R.M., Suh Y.D., Deckert V., Zenobi R. (2000). Nanoscale chemical analysis by tip-enhanced Raman spectroscopy. Chem. Phys. Lett..

[50] Xu H., Bjerneld E.J., Kall M., Borjesson L. (1999). Spectroscopy of Single Hemoglobin Molecules by Surface Enhanced Raman Scattering. Phys. Rev. Lett..

[51] Xu H., Aizpurua J., Kall M., Apell P. (2000). Electromagnetic Contributions to Single-Molecule Sensitivity in Surface-Enhanced Raman Scattering. Phys. Rev. E.

[52] Klingsporn J.M., Jiang N., Pozzi E.A., Sonntag M.D., Chulhai D., Seideman T., Jensen L., Hersam M.C., Van Duyne R.P. (2014). Intramolecular Insight into Adsorbate-Substrate Interactions via Low-Temperature, Ultrahigh-Vacuum Tip-Enhanced Raman Spectrocopy. J. Am. Chem. Soc..

[53] Touzalin T., Dauphin A.L., Joiret S., Lucas I.T., Maisonhaute E. (2016). Tip-Enhanced Raman Spectroscopy Imaging of Opaque Samples in Organic Liquid. Phys. Chem. Chem. Phys..

[54] Kurouski D., Mattei M., Van Duyne R.P. (2015). Probing Redox Reactions at the Nanoscale with Electrochemical Tip-Enhanced Raman Spectroscopy. Nano Lett..

[55] Zeng Z.C., Huang S.C., Wu D.Y., Meng L.Y., Li M.H., Huang T.X., Zhong J.H., Wang X., Yang Z.L., Ren B. (2015). Electrochemical Tip-Enhanced Raman Spectroscopy. J. Am. Chem. Soc..

[56] Sabanes N.M., Driessen L.M.A., Domke K.F. (2016). Versatile Side-Illumination Geometry for Tip-Enhanced Raman Spectroscopy at Solid/Liquid Interfaces. Anal. Chem..

[57] Hartschuh A., Qian H., Meixner A.J., Anderson N., Novotny L. (2006). Nanoscale Optical Imaging of Single-Walled Carbon Nanotubes. J. Lumin..

[58] Hartschuh A., Anderson N., Novotny L. (2003). Near-Field Raman Spectroscopy using a Sharp Metal Tip. J. Microsc..

[59] Anger P., Bharadwaj P., Novotny L. (2006). Enhancement and Quenching of Single-Molecule Fluorescence. Phys. Rev. Lett..

[60] Neacsu C.C., Berweger S., Raschke M.B. (2007). Tip-enhanced Raman imaging and nanospectroscopy: Sensitivity, symmetry, and selection rules. Nanobiotechnology.

[61] Lloyd J.S., Williams A., Rickman R.H., McCowen A., Dunstan P.R. (2011). Reproducible electrochemical etching of silver probes with a radius of curvature of 20 nm for tip-enhanced Raman applications. Appl. Phys. Lett..

[62] Yang Z., Aizpurua J., Xu H. (2009). Electromagnetic field enhancement in TERS configurations. J. Raman Spectrosc..

[63] Festy F., Demming A., Richards D. (2004). Resonant excitation of tip plasmons for tipenhanced Raman SNOM. Ultramicroscopy.

[64] Yeo B.S., Zhang W., Vannier C., Zenobi R. (2006). Enhancement of Raman signals with silver-coated tips. Appl. Spectrosc..

[65] Kharintsev S.S., Hoffmann G.G., Dorozhkin P.S., de With G., Loos J. (2007). Atomic force and shear force based tip-enhanced Raman spectroscopy and imaging. Nanotechnology.

[66] Johnson P.B., Christy R.W. (1972). Optical constants of the noble metals. Phys. Rev. B.

[67] Novotny L., Bian R., Xie X. (1997). Theory of nanometric optical tweezers. Phys. Rev. Lett..

[68] Mcmahon M.D., Lopez R., Meyer H.M., Feldman L.C., Haglund R.F. (2005). Rapid tarnishing of silver nanoparticles in ambient laboratory air. Appl. Phys. B.

[69] Stockle R.M., Deckert V., Fokas C., Zenobi R. (2000). Controlled formation of isolated silver islands for surface-enhanced Raman scattering. Appl. Spectrosc..

[70] Nieman L.T., Krampert G.M., Martinez R.E. (2001). An apertureless near-field scanning optical microscope and its application to surface-enhanced Raman spectroscopy and multiphoton fluorescence imaging. Rev. Sci. Instrum..

[71] Saito Y., Wang J.J., Smith D.A., Batchelder D.N. (2002). A simple chemical method for the preparation of silver surfaces for efficient SERS. Langmuir.

[72] Wang J.J., Saito Y., Batchelder D.N., Kirkham J., Robinson C., Smith D.A. (2005). Controllable method for the preparation of metallized probes for efficient scanning near-field optical Raman microscopy. Appl. Phys. Lett..

[73] Vasile M.J., Grigg D.A., Griffith J.E., Fitzgerald E.A., Russell P.E. (1991). Scanning probe tips formed by focused ion beams. Rev. Sci. Instrum..

[74] Sánchez E., Novotny L., Xie X. (1999). Near-field fluorescence microscopy based on two photon excitation with metal tips. Phys. Rev. Lett..

[75] Becker M., Sivakov V., Gösele U., Stelzner T., Andrä G., Reich H.J., Hoffmann S., Michler J., Christiansen S.H. (2008). Nanowires enabling signal-enhanced nanoscale Raman spectroscopy. Small.

[76] Becker M., Sivakov V., Andrä G., Geiger R., Schreiber J., Hoffmann S., Michler J., Milenin A.P., Werner P., Christiansen S.H. (2007). The SERS and TERS effects obtained by gold droplets on top of Si nanowires. Nano Lett..

[77] Yang Y., Li Z.-Y., Nogami M., Tanemura M., Huang Z. (2014). The controlled fabrication of “Tip-On-Tip” TERS probes. RSC Adv..

[78] Johnson T.W., Lapin Z.J., Beams R., Lindquist N.C., Rodrigo S.G., Novotny L. (2012). Highly reproducible near-field optical imaging with Sub-20-nm resolution based on template-stripped gold pyramids. ACS Nano.

[79] Fujita Y., Walke P., De Feyter S., Hiroshi Uji-i H. (2016). Remote excitation-tip-enhanced Raman scattering microscopy using silver nanowire. Jpn. J. Appl. Phys..

[80] Futamata M., Maruyama Y., Ishikawa M. (2003). Local electric field and scattering cross section of Ag nanoparticles under surface plasmon resonance by finite difference time domain method. J. Phys. Chem. B.

[81] Verma P., Yamada K., Watanabe H., Inouye Y., Kawata S. (2006). Near-field Raman scattering investigation of tip effects on C_60_ molecules. Phys. Rev. B.

[82] Treffer R., Böhme R., Deckert-Gaudig T., Lau K., Tiede S., Lin X., Deckert V. (2012). Advances in TERS (tip-enhanced Raman scattering) for biochemical applications. Biochem. Soc. Trans..

[83] Bailo E., Deckert V. (2008). Tip-enhanced Raman scattering. Chem. Soc. Rev..

[84] Deckert-Gaudig T., Bailo E., Deckert V. (2008). Perspectives for spatially resolved molecular spectroscopy—Raman on the nanometer scale. J. Biophotonics.

[85] Rasmussen A., Deckert V. (2006). Surface- and Tip-Enhanced Raman Scattering of DNA Components. J. Raman Spectrosc..

[86] Ichimura T., Hayazawa N., Hashimoto M., Inouye Y., Kawata S. (2004). Tip-Enhanced Coherent Anti-Stokes Raman Scattering for Vibrational Nanoimaging. Phys. Rev. Lett..

[87] Domke K.F., Zhang D., Pettinger B. (2007). Tip-Enhanced Raman Spectra of Picomole Quantities of DNA Nucleobases at Au(111). J. Am. Chem. Soc..

[88] Zhang D., Domke K.F., Pettinger B. (2010). Tip-Enhanced Raman Spectroscopic Studies of the Hydrogen Bonding between Adenine and Thymine Adsorbed on Au(111). ChemPhysChem.

[89] Treffer R., Lin X., Bailo E., Deckert-Gaudig T., Deckert V. (2011). Distinction of nucleobases—A tip-enhanced Raman approach. Beilstein J. Nanotechnol..

[90] Najjar S., Talaga D., Schue L., Coffinier Y., Szunerits S., Boukherroub R. (2014). Tip-enhanced Raman spectroscopy of combed double-stranded DNA bundles. J. Phys. Chem. C.

[91] Yeo B.S., Madler S., Schmid T., Zhang W.H., Zenobi R. (2008). Tip-enhanced Raman spectroscopy can see more: The case of cytochrome C. J. Phys. Chem. C.

[92] Deckert-Gaudig T., Bailo E., Deckert V. (2010). Tip-enhanced Raman scattering (TERS) and high-resolution bio nano-analysis—A comparison. Phys. Chem. Chem. Phys..

[93] Moretti M., Zaccaria R.P., Descrovi E., Das G., Leoncini M., Liberale C., De Angelis F., Di Fabrizio E. (2013). Reflection-mode TERS on insulin amyloid fibrils with top-visual AFM probes. Plasmonics.

[94] Deckert-Gaudig T., Kämmer E., Deckert V. (2012). Tracking of nanoscale structural variations on a single amyloid fibril with tip-enhanced Raman scattering. J. Biophotonics.

[95] Paulite M., Blum C., Schmid T., Opilik L., Eyer K., Walker G.C., Zenobi R. (2013). Full spectroscopic tip-enhanced Raman imaging of single nanotapes formed from β-amyloid(1–40) peptide fragments. ACS Nano.

[96] Gullekson C., Lucas L., Hewitt K., Kreplak L. (2011). Surface-sensitive Raman spectroscopy of collagen I fibrils. Biophys. J..

[97] Neugebauer U., Schmid U., Baumann K., Ziebuhr W., Kozitskaya S., Deckert V., Schmitt M., Popp J. (2007). Towards a detailed understanding of bacterial metabolism—Spectroscopic characterization of Staphylococcus epidermidis. ChemPhysChem.

[98] Cialla D., Deckert-Gaudig T., Budich C., Laue M., Moller R., Naumann D., Deckert V., Popp J. (2009). Raman to the limit: Tip-enhanced Raman spectroscopic investigations of a single tobacco mosaic virus. J. Raman Spectrosc..

[99] Rusciano G., Zito G., Isticato R., Sirec T., Ricca E., Bailo E. (2014). Nanoscale Chemical Imaging of Bacillus subtilis Spores by Combining Tip-Enhanced Raman Scattering and Advanced Statistical Tools. ACS Nano.

[100] Barros E., Carvajal C. (2017). Urinary Exosomes and Their Cargo: Potential Biomarkers for Mineralocorticoid Arterial Hypertension. Front. Endocrinol..

[101] Nalejska E., Mączyńska E., Lewandowska M.A. (2014). Prognostic and Predictive Biomarkers: Tools in Personalized Oncology. Mol. Oncol. Gene..

[102] Aronson J. (2005). Biomarkers and surrogate endpoints. Brit. J. Clin. Pharmacol..

